# Transmission and decorrelation methods for detecting rare variants using sequencing data from related individuals

**DOI:** 10.1186/s12919-016-0031-z

**Published:** 2016-10-18

**Authors:** Burcu F. Darst, Corinne D. Engelman

**Affiliations:** 1 University of Wisconsin, Madison, WI USA; 2 Department of Population Health Sciences, University of Wisconsin School of Medicine and Public Health, Madison, WI USA

## Abstract

**Background:**

Advances in whole genome sequencing have enabled the investigation of rare variants, which could explain some of the missing heritability that genome-wide association studies are unable to detect. Most methods to detect associations with rare variants are developed for unrelated individuals; however, several methods exist that utilize family studies and could have better power to detect such associations.

**Methods:**

Using whole genome sequencing data and simulated phenotypes provided by the organizers of the Genetic Analysis Workshop 19 (GAW19), we compared family-based methods that test for associations between rare and common variants with a quantitative trait. This was done using 2 fairly novel methods: family-based association test for rare variants (FBAT-RV), which is a transmission-based method that utilizes the transmission of genetic information from parent to offspring; and Minimum *p* value Optimized Nuisance parameter Score Test Extended to Relatives (MONSTER), which is a decorrelation method that instead attempts to adjust for relatedness using a regression-based method. We also considered family-based association test linear combination (FBAT-LC) and FBAT-Min P, which are slightly older methods that do not allow for the weighting of rare or common variants, but contrast some of the limitations of FBAT-RV.

**Results:**

MONSTER had much higher overall power than FBAT-RV and FBAT-Min P. Interestingly, FBAT-LC had similar overall power as MONSTER. MONSTER had the highest power for a gene accounting for a larger percent of the phenotypic variance, whereas MONSTER and FBAT-LC both had the highest power for a gene accounting for moderate variance. FBAT-LC had the highest power for a gene accounting for the least variance.

**Conclusions:**

Based on the simulated data from GAW19, MONSTER and FBAT-LC were the most powerful of the methods assessed. However, there are limitations to each of these methods that should be carefully considered when conducting an analysis of rare variants in related individuals. This emphasizes the need for methods that can incorporate the advantages of each of these methods into 1 family-based association test for rare variants.

## Background

As sequencing technologies continue to improve, new opportunities arise to detect rare variants in complex human traits. Genome-wide association studies (GWAS) have been able to detect thousands of markers associated with various traits [1]. However, these markers generally have common alleles (minor allele frequency >5 %) and small effects. Advances in whole genome sequencing (WGS) have enabled the investigation of rare variants, which could potentially explain some of the missing heritability that GWAS are unable to detect [2, 3].

Until recently, methods for the analysis of rare variants typically focused on unrelated individuals. However, family-based studies may be better powered to detect rare variants because of their potential to be enriched for rare variants [4]. Family-based studies are also advantageous because they can be robust to population stratification when calculating within family statistics, facilitate the detection of sequencing errors, and allow investigators to test complex hypotheses, such as parent-of-origin effects [5].

Using WGS data and simulated phenotypes provided for Genetic Analysis Workshop 19 (GAW19) [6], we compared family-based methods that test for associations between rare and common variants with a quantitative trait. This was done using the family-based association test for rare variants (FBAT-RV), which is a transmission-based method that utilizes the transmission of genetic information from parent to offspring [7], and Minimum *p* value Optimized Nuisance parameter Score Test Extended to Relatives (MONSTER), which is a decorrelation method that instead attempts to adjust for relatedness using a regression-based method [8]. We also considered FBAT linear combination (FBAT-LC) [9] and FBAT-Min P [10], which are slightly older methods that do not allow for the weighting of rare or common variants, but which contrast some of the limitations of FBAT-RV. Analyses were conducted with knowledge of the simulation model.

## Methods

### Data description

The data sets provided consisted of family-based WGS data and 200 replicates of simulated phenotypes. WGS data were provided for 959 individuals, of which 464 individuals were sequenced, while the rest were imputed. We removed 4 pedigrees where no individuals passed quality control (QC) in the first phase of sequencing (*n =* 146), leaving a total of 813 individuals in 16 families. Of these 813 individuals, we used the 719 for whom simulated phenotypic data existed.

The primary phenotypes of interest in this study were diastolic blood pressure (DBP) and a quantitative variable with a null association, Q1. DBP was simulated to have an association with several variants and is used here to assess power, while Q1 facilitates the assessment of type 1 error. To allow for the most optimal association results, in this analysis DBP was adjusted for antihypertensive medication use, sex, age, and sex*age, while Q1 was adjusted for sex and age, which is consistent with how the data were simulated. Analyses were based on the first time point and were replicated using the provided 200 phenotypic simulation data sets. We focused on 3 of the top genes that explained the most variance in the simulated DBP variable: *MAP4* (in chromosome 3), *TNN*, and *LEPR* (both in chromosome 1).

### Annotations and quality control

Gene-based annotations were performed using ANNOVAR (Annotate Variation) [11] and the human genome RefSeq database based on hg19. Intergenic sites within 5 kbp of a gene were mapped to the closest gene. Those that were further than 5 kbp from a gene were excluded, as the simulation model selected causal variants that were within this range. In addition to the QC measures taken by the organizers of GAW before releasing the data, further QC steps were taken using VCFtools version 0.1.12a [12]. Sites with a call rate of less than 95 % and sites that were out of Hardy-Weinberg equilibrium within the 91 founders were removed. This resulted in 850 sites in *MAP4*, 493 in *TNN*, and 899 in *LEPR*.

### Rare variant analysis methods

The transmission-based rare variant analyses were conducted using FBAT-RV, FBAT-LC, and FBAT-Min P. FBAT-RV extends the basic family-based association test (FBAT) statistic, a covariance between the offspring genotype and trait, by collapsing rare variants over a specified region, resulting in a test statistic for that region [7]. FBAT-RV builds on the FBAT multi-marker test, which is a gene-based test for family studies assessing multiple variants in candidate genes [13]. We used FBAT-RV’s weighted method, as a previous study found that it is generally more powerful than the unweighted method [14]. The weighted method allows for the inclusion of both common and rare variants by up-weighting rarer variants and down-weighting common variants using the following weight, ${w}_s={\left(\sqrt{n{p}_s\left(1-{p}_s\right)}\right)}^{-1}$, where *n* is the total number of nuclear families and *p*_*s*_ is the allele frequency for the *s* ^*th*^ variant estimated from the sample. This weighting method is similar to that of Madsen and Browning [15] and estimates allele frequencies using the parents.

FBAT-LC and FBAT-Min P are both FBAT multi-marker tests and use an empirical variance–covariance matrix to estimate the covariance between the markers. FBAT-LC uses non-informative families to estimate the optimal weights for the linear combination of the single-marker test statistics [9]. FBAT-Min P uses the Monte Carlo permutation to obtain a *p* value for the maximally significant statistic out of the set of individual statistics [10]. These 2 tests were chosen because they are able to handle variants that have effects in the opposite direction, unlike FBAT-RV. However, these tests were not designed for the analysis of rare variants and do not allow for weighting based on allele frequency.

Because multiple families were in each pedigree, all 3 FBAT tests were computed using the empirical variance option (–e) suggested in the FBAT documentation. The 2 outcomes, DBP and Q1, were adjusted for the aforementioned covariates of interest by putting them into linear regression models and using the resulting residuals as the outcome in the FBAT models.

The decorrelation-based rare variant analysis was conducted using MONSTER [7]. This test uses a hierarchical-mixed effects model and is considered to be an extension of SKAT-O and a convex combination of famSKAT and famBT, either mimicking or improving the performance of the 2 programs. MONSTER assumes that pedigree information is known and accounts for relatedness using kinship coefficients for all possible pairs of individuals within each family. Here, we estimated theoretical pedigree-based kinships using the KinInCoef software [16], although it is possible to use empirical kinships. Each outcome was adjusted for covariates within MONSTER by adding the variables to the model. Similar to FBAT-RV, variants were collapsed by gene and weighted using the beta distribution density function described in Wu et al with a_1_ = 1 and a_2_ = 25 [17].

Collapsing methods, using the gene plus 5 kbp on either side as the collapsed region, were employed for each of these approaches, as it has been found that power is increased when the effects of multiple rare variants are combined [18]. Computation was performed on a 64-bit Linux server cluster.

### Power and type 1 error

All 4 methods were tested with genes that had a known association with DBP and a null association with Q1, based on the GAW19 simulation model. Each method was repeated using each of the simulated replicates, resulting in 200 *p* values for each gene. Type 1 error was defined as the proportion of *p* values under 0.05 for each gene when tested with Q1, which was then averaged across all genes to estimate the type 1 error rate of the method. Power to detect an association between each gene and DBP was defined as the proportion of *p* values under a threshold that made type I error equal to exactly 0.05. This was similarly averaged across all genes to estimate the overall power of the method.

## Results

Type I error rates and power and for all 4 methods used are described in Tables 1 and 2, respectively. Table 2 also describes the percentage of variance explained by each gene and the number of functional single nucleotide polymorphisms (SNPs) in each based on the simulation model, as described by Almasy et al [6]. Type I error rates were lowest for FBAT-Min P and highest in FBAT-LC. Holding type I error at 0.05, MONSTER had much higher overall power than FBAT-RV and FBAT-Min P (44 % compared to 24 % and 19 %, respectively). Interestingly, FBAT-LC had similar overall power to MONSTER.

**Table 1:** Type I error rates (Q1 trait)

	MONSTER	FBAT		
		RV	LC	Min P
*MAP4*	0.04	0.03	0.04	0.01
*TNN*	0.065	0.04	0.06	0
*LEPR*	0.075	0.055	0.09	0.005
Average	0.06	0.04	0.07	0.005

Type I error rates at an alpha level of 0.05

**Table 2:** Genetic variance and power (DBP)

	# SNPs	# Fn. SNPs^a^	% Var. Explained^a^	% Var. of Largest Fn. Variant^a^	MONSTER	FBAT		
						RV	LC	Min P
*MAP4*	919	15	6.48	2.29	1.00	0.57	0.82	0.37
*TNN*	493	18	4.08	1.98	0.31	0.12	0.31	0.13
*LEPR*	899	8	2.50	2.19	0.03	0.05	0.16	0.08
Average	770.3	13.7	4.35	1.78	0.44	0.24	0.43	0.19
Computation time (min)					39	13	21	52

*Fn* functional, *Var* variance

Power is calculated using a type I error of 0.05

^a^Data on the number of SNPs simulated to be functional and the percent of variance explained by these functional SNPs has been provided by Almasy et al. [23]

Results from MONSTER showed that each replicate was able to detect an association between DBP and *MAP4* (power = 100 %), which was simulated to explain the highest amount of variance in DBP. However, it did not perform quite as well for *TNN* or *LEPR*, the latter of which had particularly low power. FBAT-LC had similar overall performance to MONSTER and outperformed FBAT-RV for all 3 genes; FBAT-RV had moderate power for *MAP4*, but poor power for *LEPR* and *TNN*. However, FBAT-RV did have slightly lower type 1 error rates than both MONSTER and FBAT-LC for each gene and overall. FBAT-Min P had the lowest overall power, but performed similarly to FBAT-RV for *TNN* and *LEPR.* As would be expected, Tables 1 and 2 show that for all tests, as effect sizes decreased, the power to detect an association decreased and, with the exception of FBAT-Min P, the type 1 error rate increased. MONSTER appeared to have the best performance for genes with larger effects, while MONSTER and FBAT-LC both had the best performance for genes with moderate effects. FBAT-LC had the best performance for genes with smaller effects, but even so, this power was fairly low. FBAT-RV had the quickest computation time, followed by MONSTER and FBAT-LC, with FBAT-Min P having the longest computation time (Table 2).

## Discussion

Using family-based data provided by GAW19, we evaluated the power of a decorrelation-based test and several transmission-based tests to detect associations between simulated quantitative phenotypes and rare and common variants in the *MAP4*, *TNN*, and *LEPR* genes. We found that MONSTER and FBAT-LC had superior performance when compared to FBAT-RV and FBAT-Min P. Although MONSTER and FBAT-LC had similar overall power, when considering each gene separately, they actually performed quite differently. Although MONSTER had much higher power for the *MAP4* gene, which explained the highest percent of variance of the genes assessed, FBAT-LC had higher power for the *LEPR* gene, which explained the lowest percent of variance. While FBAT-RV and MONSTER are 2 relatively novel approaches designed for the analysis of rare genetic variants within families, FBAT-LC was not intended for the analysis of rare variants. However, our results, along with those of Zhou et al [19], indicate that FBAT-LC may be useful in the detection of associations with rare variants. Our results also consistently showed that as the percentage of variance explained by each gene decreased, power to detect an association notably decreased. Although more efficient approaches should be developed to increase power, rare variants with small effect sizes will remain difficult to detect.

Computationally, we found benefits to both MONSTER and FBAT. Although FBAT-RV does have a built-in weighting method, MONSTER allows user-defined weights, which can be desirable. Neither FBAT-LC nor FBAT-Min P provides options for use of weights. A practical advantage of MONSTER is that it allows the user to enter multiple genes into a single run and then collapses them accordingly, while FBAT-RV, FBAT-LC, and FBAT-Min P require a separate run for each gene. However, the computation time for FBAT-RV was notably shorter than all other methods, with MONSTER taking 3 times longer to run.

One limitation of MONSTER is that it adjusts for the pedigree-based estimation of kinship, which is not robust against population stratification. This could explain why the MONSTER results tended to have slightly elevated false-positive rates [20]. However, MONSTER could be extended to better adjust for population stratification by using the empirical kinship correlation matrix calculated from genome-wide SNP data. FBAT is robust to population stratification, but does not consider between-family information, which could explain why the FBAT tests generally had lower power than MONSTER. Recently, a new method was developed that addresses both of these issues by integrating the QTDT (quantitative transmission disequilibrium test) framework [21] into the kernel based model, KMFAM [22]. Another possible explanation of the differences seen between FBAT and MONSTER is that FBAT only considers nuclear families, whereas MONSTER adjusts for extended family members using a kinship coefficient. This is a particularly important feature for the analysis of rare variants, which will be shared by chance in about half of all siblings in a family, but in a much lower percent of extended family members, thus reducing false-positive associations. For example, first cousins, who share, on average, 12.5 % of their DNA, can be more informative if they both have a rare variant that increases the risk for a trait and the trait itself than siblings, who share approximately 50 % of their DNA. In siblings, the association may be lost in the vast number of shared variants. An additional limitation of FBAT-RV is that it cannot handle variants within the collapsed unit that have effects in the opposite direction, unlike MONSTER, FBAT-LC, and FBAT-Min P. Although the top 55 variants in the GAW19 simulation model had effects in the same direction within a gene [23], some of the variants with smaller effects may have been in the opposite direction. This limitation should be taken into consideration as it could occur in real data.

Saad and Wijsman recently reported that using allelic dosages from imputation as opposed to genotypes leads to higher power [24]. MONSTER can utilize data files with allelic dosages, and a recent version of FBAT also handles dosages (FBAT-dosage [25]). However, FBAT is currently unable to handle both dosages and rare variants, and as a result, our analyses were based on genotypes, which likely reduced the power to detect associations for all the methods. We also were unable to utilize the longitudinal data provided by GAW19 because of limitations of these 2 programs. MONSTER is not currently equipped to handle longitudinal traits but FBAT has several approaches that allow for the analysis of longitudinal data, one of which being FBAT-LC. Because our results suggest that FBAT-LC may be an appropriate method to detect associations with rare variants, this test could potentially be used for the analysis of rare variants and longitudinal data.

## Conclusions

Based on the simulated data from GAW19, MONSTER and FBAT-LC were the most powerful of the methods assessed. However, even these methods suffer from low power as the amount of variance explained by genes decreased. Furthermore, there are limitations to each of these methods that should be carefully considered when conducting an analysis of rare variants in related individuals. This study emphasizes the need for more efficient FBATs that can incorporate the advantages of each of the tests assessed and increase the power to detect associations for rare variants with moderate to low effects.
